# USP10 modulates the SKP2/Bcr-Abl axis via stabilizing SKP2 in chronic myeloid leukemia

**DOI:** 10.1038/s41421-019-0092-z

**Published:** 2019-04-30

**Authors:** Yuning Liao, Ningning Liu, Xiaohong Xia, Zhiqiang Guo, Yanling Li, Lili Jiang, Ruiqing Zhou, Daolin Tang, Hongbiao Huang, Jinbao Liu

**Affiliations:** 1 Affiliated Cancer Hospital and institute of Guangzhou Medical University; Key Laboratory of Protein Modification and Degradation, State Key Laboratory of Respiratory Disease, School of Basic Medical Sciences, Guangzhou Medical University, Guangzhou, Guangdong 511436 China; 2 Guangzhou Institute of Cardiovascular Disease, the Second Affiliated Hospital, Guangzhou Medical University, Guangzhou, Guangdong 510260 China; 3 Department of Hematology, Guangzhou First People’s Hospital, Guangzhou Medical University, Guangzhou, Guangdong 511436 China

**Keywords:** Chronic myeloid leukaemia, Drug development

## Abstract

Constitutive activation of tyrosine kinase Bcr-Abl is the leading cause of the development and progression of chronic myeloid leukemia (CML). Currently, the application of tyrosine kinase inhibitors (TKIs) targeting the Bcr-Abl is the primary therapy for CML patients. However, acquired resistance to TKIs that develops overtime in the long-term administration renders TKIs ineffective to patients with advanced CML. Therefore, increasing studies focus on the amplified expression or activation of Bcr-Abl which is proposed to contribute to the advanced phase. Here, we show that S-phase kinase-associated protein 2 (SKP2) acts as a co-regulator of Bcr-Abl by mediating its K63-linked ubiquitination and activation. Further investigations show that USP10 as a novel deubiquitinase of SKP2 amplifies the activation of Bcr-Abl via mediating deubiquitination and stabilization of SKP2 in CML cells. Moreover, inhibition of USP10 significantly suppresses the proliferation of both imatinib-sensitive and imatinib-resistant CML cells, which likely depends on SKP2 status. This findings are confirmed in primary CML cells because these cells are over-expressed with USP10 and SKP2 and are sensitive to a USP10 inhibitor. Taken together, the present study not only provides a novel insight into the amplified activation of Bcr-Abl in CML, but also demonstrates that targeting the USP10/SKP2/Bcr-Abl axis is a potential strategy to overcome imatinib resistance in CML patients.

## Introduction

Chronic myeloid leukemia (CML), a hematopoietic malignancy, is caused by constitutively high autophosphorylation of the oncogenic tyrosine kinase Bcr-Abl that results from the translocation of t(9;22)(q34;q11)^1,2^. Bcr-Abl contributes to the tumorigenesis of CML by activating multiple signaling pathways, including MAPK/ERK, PI3K/Akt, JNK, STAT5, and SRC^3–7^. Subsequent evidence shows that Bcr-Abl may promote the transition of G1 to S phase by enhancing the transcription and inhibiting the degradation of S-phase kinase-associated protein 2 (SKP2)^8–10^. CML cells were considered to be equipped with the ability of apoptosis escape and immortalization by the activation of these signaling pathways^11–13^. Imatinib mesylate (IM or STI571), the first selective tyrosine kinase inhibitor (TKI), has not only displayed a high efficacy in the treatment of CML patients but also provided a classical tool for studying the pathological activities of Bcr-Abl kinase^9,14,15^. Unfortunately, the long-term administration of IM in CML patients was limited by acquired resistance that develops over time^16^. Among the mechanisms underlying CML resistance to imatinib, the T315I mutation of Bcr-Abl is the most notorious; even the most potent TKIs are ineffective to patients with the T315I mutation because this mutation impedes the access of IM to the active pocket of Bcr-Abl kinase^17–19^. Therefore, overcoming T315I mutation is considered the greatest challenge in CML treatment^20–23^.

SKP2 is an F-box protein which can team up with Skp1 and Cullin1 to form a Skp1-Cullin1-F-box (SCF) type ubiquitin E3 ligase complex known as SCF^SKP2^. An early study demonstrated that SKP2 promotes G1 to S phase transition via enhancing ubiquitin-dependent proteasomal degradation of p27^24^. Recent studies showed that SKP2 promotes tumorigenesis via mediating the ubiquitination of FOXO1^25^, and Akt^26^, or suppressing cellular senescence^27^. Importantly, SKP2 is frequently overexpressed in human cancers^28,29^. SKP2 may also promote leukemogenesis^27^. SKP2 is therefore proposed as a promising target for suppressing multiple cancers. However, the roles of SKP2 and mechanisms regulating its protein level in CML remain largely unclear.

Ubiquitin-specific peptidase 10 (USP10) is a member of mammalian deubiquitinases (DUBs). It is well documented that DUBs mediate the ubiquitin cycle via removing the ubiquitin/ubiquitin chain from their specific substrates^30^, and therefore being involved in the regulation of most cellular processes^31^. However, the pathophysiology of USP10 in humans has not been fully validated. But several studies revealed that USP10 plays multiple roles in caners via regulating different substrates, including androgen receptor^32,33^, p53^34^, SIRT6^35^, AMPK^36^, and FLT3^37^. To date, the roles of USP10 in resistant CML remains unknown. The present study unraveled that USP10 stabilizes SKP2 and thereby increases Bcr-Abl activation in CML. The USP10/SKP2/Bcr-Abl may represent a druggable target for resistant CML patients with the T315I mutation.

## Results

### SKP2 interacts with Bcr-Abl and is required for the activation of Bcr-Abl

**Fig. 1:**
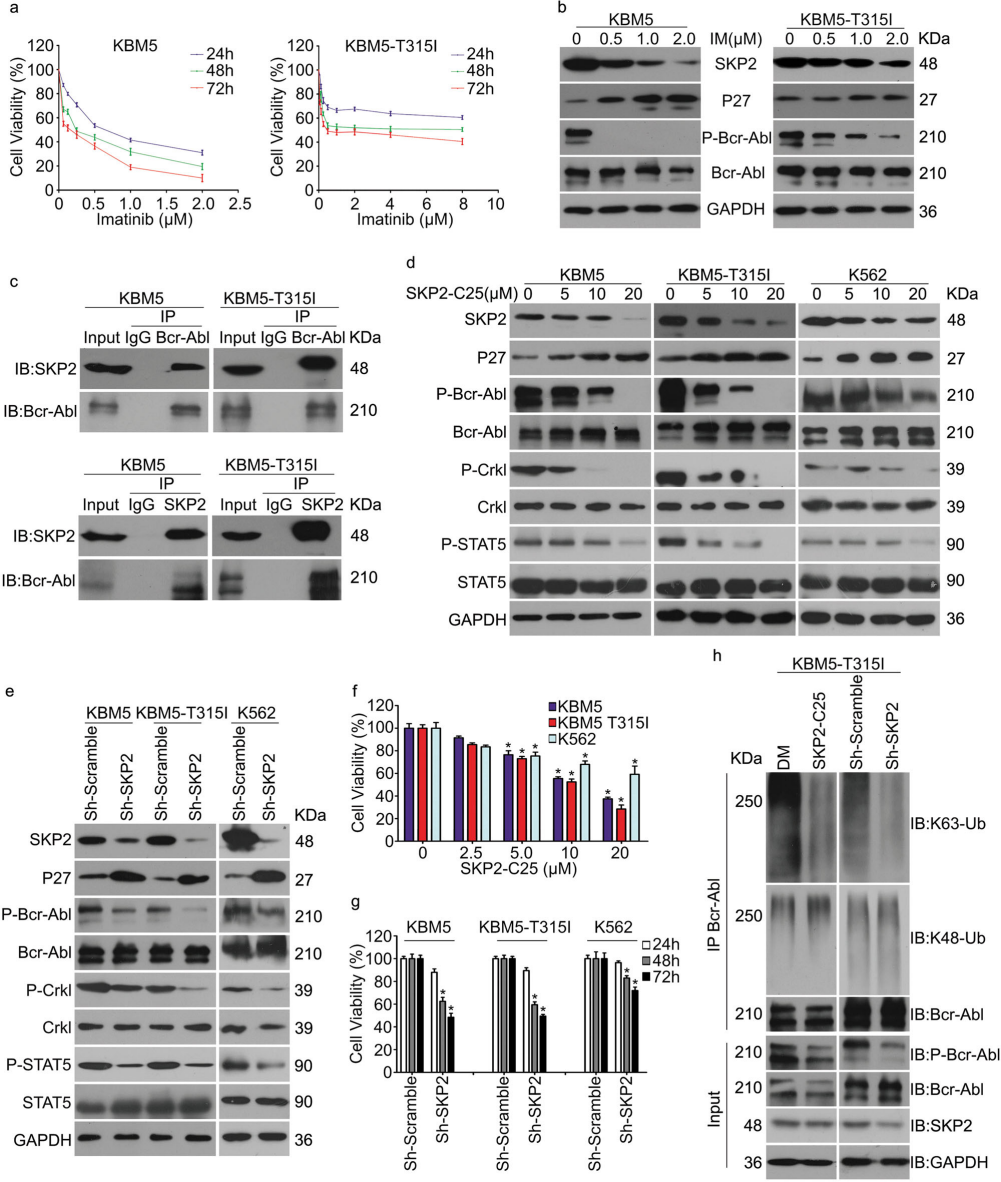
SKP2 interacts with, and facilitates the activation of, Bcr-Abl in CML cells. **a** Cell viability of CML cells post Imatinib (IM) treatment for the indicated times. **b** Western blot analysis for the indicated proteins in CML cells exposed to IM for 24 h. **c** Western blot analysis and Co-IP analysis for the indicated proteins in CML cells. **d**, **e** Western blot assay for the indicated proteins in CML cells exposed to specific inhibitor of SKP2 (SKP2-C25) or stably expressing SKP2 shRNA. **f**, **g** Cell viability of CML cells exposed to SKP2-C25 for 48 h or stably expressing SKP2 shRNA/control shRNA for 24, 48, and 72 h. Error bars correspond to 95% confidence intervals of three independent experiments. *P < 0.05 versus each vehicle control. **h** Western blot and Co-IP assays to detect K63/K48-linked ubiquitination of Bcr-Abl of KBM5-T315I cells post SKP2 shRNA/SKP2-C25 treatment

Previous studies have shown that SKP2 is downstream of Bcr-Abl and promotes CML cell proliferation^8–10^. On one hand, Bcr-Abl enhances mRNA expression of SKP2 via the PI3K/AKT/Sp1 pathway^9^; on the other hand, Bcr-Abl inhibits APC/Cdh1 to stabilize SKP2 via inducing tyrosine phosphorylation of Emi1, a negative regulator of the APC/Cdh ligase^10^. Initially, we verified this interaction in two typical CML cell lines: the wild-type KBM5 and KBM5-T315I that bears the T315I mutation. Notably, inhibition of Bcr-Abl kinase by IM led to significant decrease of the expression of SKP2 and increase of the expression of p27. Notably, compared to KBM5-T315I, KBM5 cells were more sensitive to IM treatment, and the decreased phosphorylation levels of Bcr-Abl and SKP2 protein level by IM was more dramatic than that in KBM5-T315I cells, suggesting that the alteration of SKP2 is consistent with the level of phosphorylated Bcr-Abl (Fig. 1a, b). We wondered whether the biological activity of SKP2 is in turn required for Bcr-Abl. Therefore, we examined the protein interaction between Bcr-Abl and SKP2 using the Co-IP assay. As shown in Fig. 1c, we unexpectedly found that SKP2 interacted with Bcr-Abl in both IM-sensitive KBM5 and IM-resistant KBM5-T315I cells, and the interaction was not influenced by IM (Supplementary Fig. S1a), suggesting this interaction is independent of the phosphorylation of Bcr-Abl and SKP2 may have an additional role in regulating Bcr-Abl activity.

To further examine the effect of SKP2 on Bcr-Abl in vivo, we tested the effect of genetic or pharmacological inhibition of SKP2 on the phosphorylation and overall expression of Bcr-Abl in CML cells with SKP2 shRNA or SKP2-C25, a potent and specific inhibitor of SKP2^38^. Interestingly, as shown in Fig. 1d, e, we found that both genetic and pharmacological inhibition of SKP2 significantly decreased the level of phosphorylated Bcr-Abl and its downstream signals, including phosphorylated Crkl and STAT5, while total Bcr-Abl expression was not affected in all cells examined. Moreover, because the phosphorylation level of Bcr-Abl at Y245 residue in the SH2-kinase domain is critical for it’s full activation^39,40^, suggesting that SKP2 is involved in the activation of Bcr-Abl, regardless of the T315I mutation. Consistent with this hypothesis, we found that inhibition or loss of SKP2 significantly suppresses the cell viability of all K562, KBM5, and KBM5-T315I cells (Fig. 1f, g). To exclude potential off-target effects, another two pairs of SKP2 shRNAs were used to knockdown the expression of SKP2 and both SKP2 shRNAs similarly reduced the phosphorylation level of Bcr-Abl and decreased cell viability in KBM5 and KBM5-T315I cells (Supplementary Fig. S1b, c).

SKP2 is one of the E3 ligases that can mediate both K48-linked and K63-linked ubiquitination. SKP2 not only induces the K48-linked ubiquitination and proteolytic degradation of several substrates, including p27, p21, and p57^41^ but also induces the non-proteolytic K63-linked ubiquitination and activation of Akt^26^. Thus, we explored the effect of SKP2 on Bcr-Abl ubiquitination and stability. Since inhibition of SKP2 mainly affects the activity of Bcr-Abl but not increases its protein level, we hypothesized that SKP2 may not promote the proteolytic degradation of Bcr-Abl but enhance the activity of Bcr-Abl by promoting the K63-linked ubiquitination of Bcr-Abl. To examine the hypothesis, we next determined the effect of SKP2-C25 or SKP2 shRNA on the abundance of K63-linked ubiquitination and K48-linked ubiquitination levels of Bcr-Abl using Co-IP and WB. Notably, genetic or pharmacological inhibition of SKP2 decreased the K63-linked but not K48-linked ubiquitination of Bcr-Abl (Fig. 1h). Additionally, forced expression of SKP2 increased the K63-linked ubiquitination level of Bcr-Abl (Supplementary Fig. S1d). Interestingly, this treatment also raised the phosphorylation level of Bcr-Abl. Therefore, all these findings suggest that SKP2 mediates K63-linked ubiquitination of Bcr-Abl which is required for the activation of Bcr-Abl. Taken together, SKP2 not only acts as a downstream regulator of Bcr-Abl, but also a co-regulator that is critical to the activation of Bcr-Abl signaling.

### USP10 regulates SKP2 protein level and Bcr-Abl activation

**Fig. 2:**
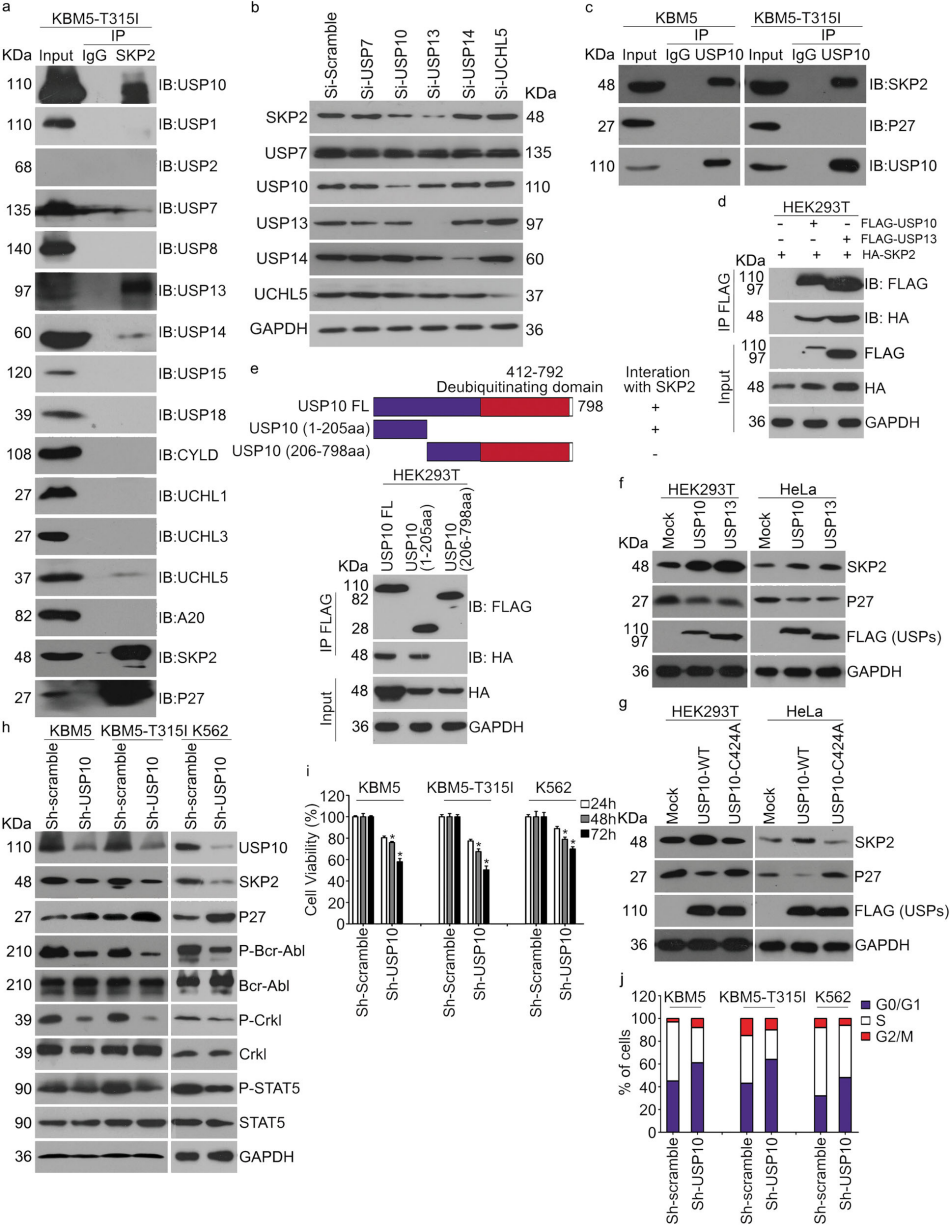
USP10 interacts with SKP2 and enhances activation of the Bcr-Abl-SKP2 axis. **a** Western blot and Co-IP assays to detect the protein interaction with SKP2. **b** Western blot assay was performed on HeLa cells post USP10, USP13, USP14, USP7, UCHL5 siRNAs or control siRNAs for 48 h. The expression of SKP2, USP10, USP13, USP14, USP7, and UCHL5 were detected. **c** Western blot and Co-IP assays to detect the protein interaction with USP10. **d** Western blot and Co-IP assays to detect the protein interaction with exogenous FLAG-USP10. **e** Mapping of USP10 and Co-IP assay shows that USP10 interacts with SKP2 through its N-terminal (1-205aa). **f** Western blot assay to detect the protein level of SKP2 and p27 of HEK293T and HeLa cells transfected with FLAG-USP10, FLAG-USP13 or control vector for 72 h. **g** Western blot assay to detect the protein level of SKP2 and p27 of HEK293T and HeLa cells transfected with FLAG-USP10 (WT), FLAG-USP10 (CA) or control vector for 72 h. **h** Western blot assay of CML cells stably expressing USP10 shRNA or control shRNA. **i** Cell viability of CML cells stably expressing USP10 shRNA or control shRNA for 24, 48, and 72 h. Error bars correspond to 95% confidence intervals of three independent experiments. *P < 0.05 versus each vehicle control. **j** Fluorescence-activated cell sorting analysis (FACS) of CML cells stably expressing USP10 shRNA or control shRNA

SKP2 protein but not its mRNA was frequently increased in some tumors^42,43^, which may result from dysregulation of post-transcriptional modification. A recent study found that USP13 regulate the Cdh1-Skp2-p27 axis in response to endoplasmic reticulum (ER) stress via deubiquitinating and stabilizing SKP2^44^. To find out whether there are other DUBs that may regulate SKP2, we screened the protein interaction between endogenous SKP2 and a panel of DUBs in CML cells by co-IP assays. We found that SKP2 interacted with USP10, USP13, UCHL5, USP14, and USP7 in KBM5-T315I cells (Fig. 2a). Given the difference of antibody affinity and western blot signals, we further detected the expression of SKP2 in HeLa cells subject to the knockdown of USP10, USP13, UCHL5, USP14 or USP7. As shown in Fig. 2b, only the knockdown of USP10 and USP13 decreased the expression of SKP2. Moreover, as shown in Fig. 2c, endogenous USP10 was found to bind SKP2 in both IM-sensitive KBM5 and IM-resistant KBM5-T315I cells. We also validated the interaction between exogenous USP10 and SKP2 using Co-IP in HEK293 cells that were co-transfected with FLAG-USP10, FLAG-USP13, and HA-SKP2 plasmids (Fig. 2d). By further mapping the regions of USP10, we showed that the N-terminal region (1-205aa) was required for the binding of USP10 to SKP2 (Fig. 2e).

We further explored whether USP10 modulates the expression of SKP2 and thus activating Bcr-Abl in CML cells. The results showed that overexpression of USP10 increased the protein level of SKP2 in HEK293T and HeLa cells in a manner similar to USP13 (Fig. 2f). To explore the requirement of deubiquitinating activity of USP10 for the enrichment of SKP2, wild type (WT) or catalytic inactive mutant (CA) USP10^34,36^ was introduced to HEK293T and HeLa cells. We further found that only USP10-WT, but not USP10-CA, increased the protein level of SKP2 (Fig. 2g), suggesting that deubiquitinating activity of USP10 is required for SKP2 enrichment. Additionally, we applied the specific shRNA of USP10 to knock down USP10 expression and tested the resultant effect on the expression levels of SKP2 and phosphorylated Bcr-Abl in K562, KBM5, and KBM5-T315I cells. The result showed that loss of USP10 significantly decreased the expression of SKP2, as well as the phosphorylation and downstream signals of Bcr-Abl (Fig. 2h), suggesting that USP10 is required for the expression of SKP2 and the phosphorylation of Bcr-Abl in CML cells.

**Fig. 3:**
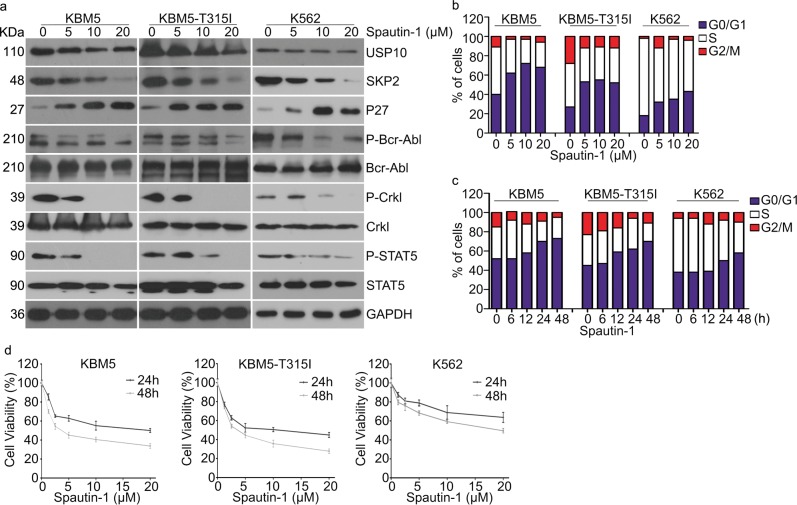
Inhibition of USP10 suppresses the activation of the Bcr-Abl-SKP2 axis and the growth of CML cells. **a** Western blot assay of indicated cell lines post Spautin-1 treatment for 48 h. **b** Fluorescence-activated cell sorting analysis (FACS) was performed to analyze the percentage of cells in each population on indicated cells exposed to Spautin-1 for 48 h. **c** Fluorescence-activated cell sorting analysis (FACS) of CML cells in each population on indicated cells exposed to Spautin-1 at different time. **d** Cell viability of indicated cell lines exposed to Spautin-1. Error bars correspond to 95% confidence intervals of three independent experiments

We next examined whether loss of USP10 could affect the proliferation or cell cycle progression of K562, KBM5, and KBM5-T315I cells. We found that loss of USP10 consequently suppressed the proliferation of CML cells and induced G0/G1 phase arrest in all CML cell lines examined (Fig. 2i, j), suggesting that USP10 is critical to CML cell proliferation. To exclude the probable off-target effects, two more pairs of USP10 shRNAs were used to knockdown the expression of USP10. As shown in supplementary Fig. S2a, b, both USP10 shRNAs similarly reduced phospho-Bcr-Abl and cell viability in both KBM5 and KBM5-T315I cells. Additionally, we also evaluated the effect of Spautin-1, a selective inhibitor of USP10 and USP13^45^, on the activation of the Bcr-Abl-SKP2 axis and other downstream signals in human CML cells. Notably, Spautin-1 significantly decreased the expression of SKP2 and activation of Bcr-Abl (Fig. 3a). Furthermore, we evaluated the effect of Spautin-1 on the cell cycle progression or cell death induction of CML cells. The results showed that Spautin-1 significantly arrested CML cells at the G0/G1 phase (Fig. 3b, c), but not apoptosis (Supplementary Fig. S3) in K562, KBM5, and KBM5-T315I cells, suggesting that Spautin-1 triggers cell line-dependent events in different tumors. The MTS results also showed that pharmacological inhibition of USP10 with Spautin-1 notably decreased the cell viability of CML cells (Fig. 3d). These data collectively suggest that USP10 is involved in the regulation of G1 to S phase transition via targeting the Bcr-Abl-SKP2-p27 axis.

### USP10 deubiquitinates and stabilizes SKP2

**Fig. 4:**
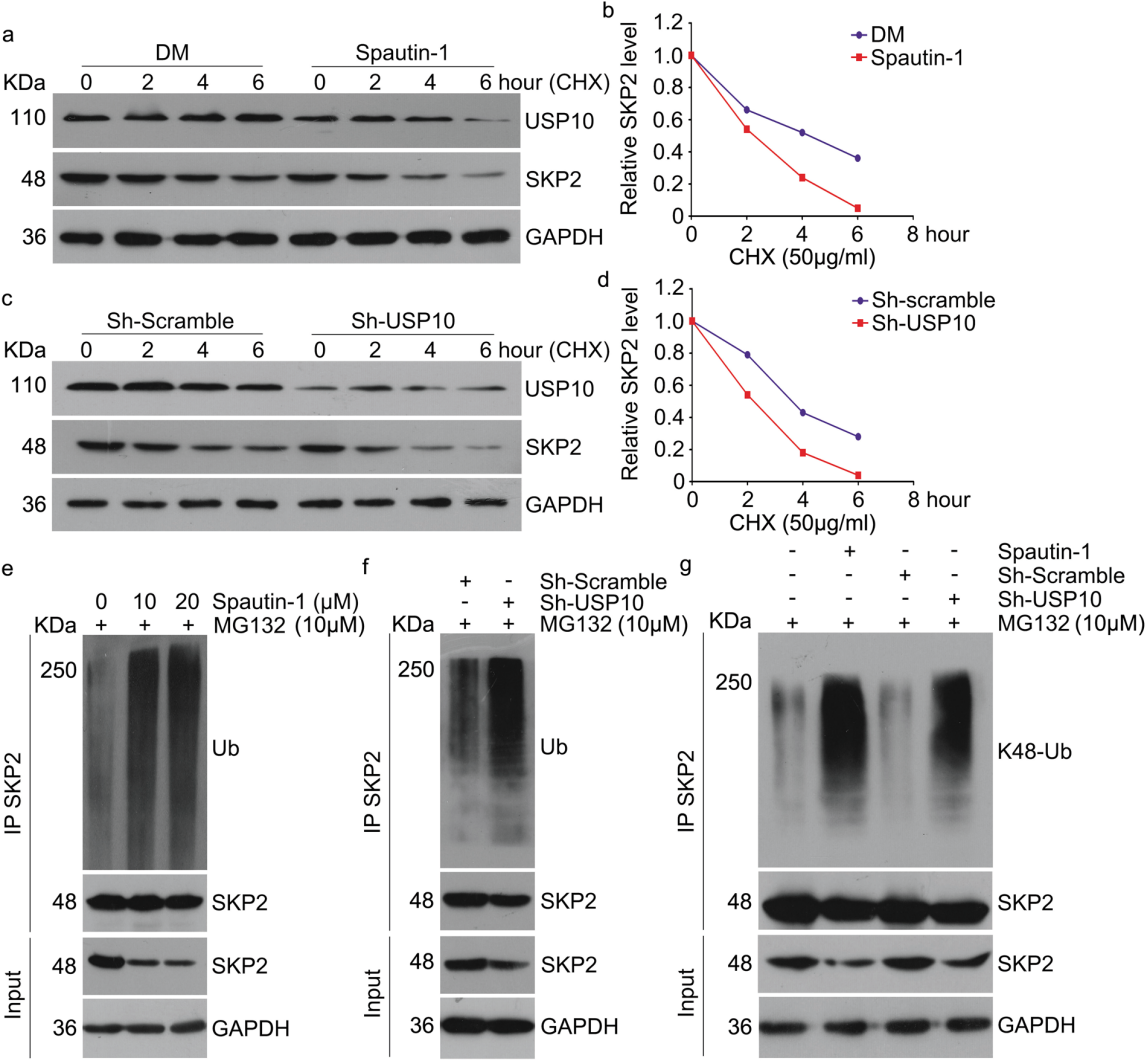
USP10 inhibits the ubiquitination and degradation of SKP2. **a** Western blot assay to detect the protein level of SKP2 in KBM5-T315I cells treated with cycloheximide (CHX) for various lengths of time with or without Spautin-1 pre-treatment for 24 h. **b** Summary of SKP2 protein levels were shown. **c** Western blot assay was used to detect the protein level of SKP2 in KBM5-T315I cells stably expressing USP10 shRNA or control shRNA treated with cycloheximide (CHX) for various lengths of time. **d** Summary of SKP2 protein levels was shown. **e** KBM5-T315I cells exposed to Spautin-1 for 48 h, immunoprecipitated with SKP2 antibodies-coupled beads, and immunoblotted for Ub and SKP2. Cells were treated with MG132 (10 μM) for 6 h before harvest. **f** KBM5-T315I cells stably expressing USP10 shRNA or control shRNA, immunoprecipitated with SKP2 antibodies-coupled beads, and immunoblotted for Ub and SKP2. Cells were treated with MG132 (10 μM) for 6 h before harvest. **g** KBM5-T315I cells exposed to Spautin-1 for 48 h or stably expressing USP10 shRNA, immunoprecipitated with SKP2 antibodies-coupled beads, and immunoblotted for K48-Ub and SKP2. Cells were treated with MG132 (10 μM) for 6 h before harvest

Since USP10 is a well-characterized DUB in humans, exploring whether USP10 directly regulates the stability of SKP2 protein is attractive. We therefore examined the effect of USP10 inhibition on endogenous SKP2 protein levels of KBM5-T315I cells in the presence of cycloheximide (CHX) which inhibits protein synthesis. Notably, genetic or pharmacological inhibition of USP10 using Spautin-1 prominently decreased the stability of endogenous SKP2 protein (Fig. 4a–d), suggesting that USP10 inhibits the degradation of SKP2 in vivo. Therefore, we wondered whether USP10 also functions as a deubiquitinase of SKP2. To our expectation, pharmacological inhibition of USP10 by Spautin-1 significantly increased the poly-ubiquitination level of SKP2 (Fig. 4e). Additionally, genetic silencing of USP10 by its specific shRNA also enriched the poly-ubiquitination levels of SKP2 (Fig. 4f). To find out whether USP10 mediated deubiquitination of SKP2 prevents its degradation, we examined the effect of Spautin-1 or USP10 shRNA on the abundance of K48-poly-ubiquitinated SKP2. We found that both Spautin-1 and USP10 shRNA dramatically enriched the abundance of K48-poly-ubiquitinated SKP2 (Fig. 4g). Collectively, these results suggest that USP10 can act as a DUB of SKP2 and thereby stabilizes SKP2 in CML cells.

### Proliferation inhibition by loss of USP10 depends on SKP2 functional status

**Fig. 5:**
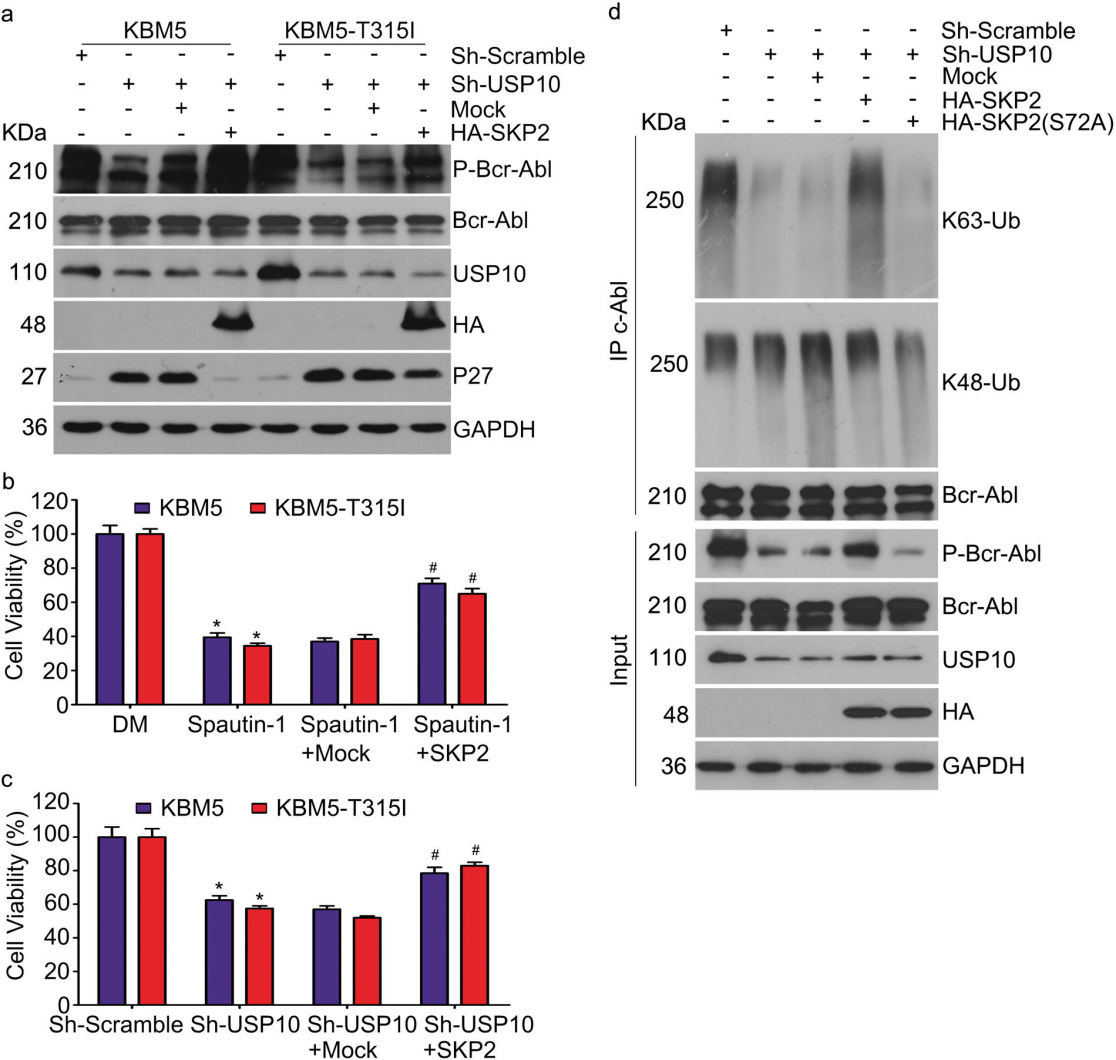
Inactivation of Bcr-Abl by USP10 inhibition or knockdown depends on SKP2 status. **a** Western blot assay of indicated CML cells stably expressing USP10 shRNA or control shRNA, with or without HA-SKP2 for 72 h. **b** Cell viability of indicated CML cells exposed to Spautin-1(10 μM) with or without HA-SKP2 for 72 h. Error bars correspond to 95% confidence intervals of three independent experiments. *P < 0.05 versus vehicle control. ^#^*P* < 0.05 versus Spautin-1 + Mock. **c** Cell viability of indicated CML cells stably expressing USP10 shRNA or control shRNA, with or without HA-SKP2 for 72 h. Error bars correspond to 95% confidence intervals of three independent experiments. *P < 0.05 versus vehicle control. ^#^*P* < 0.05 versus Sh-USP10 + Mock. **d** Total proteins were extracted from KBM5-T315I cells stably expressing USP10 shRNA or control shRNA, with or without HA-SKP2, HA-SKP2 (S72A) for 72 h, immunoprecipitated with c-Abl antibodies-coupled beads, and immunoblotted for K63-Ubiquitin (Ub), K48-Ubiquitin (Ub) and Bcr-Abl

To further determine the dependence of the USP10 function on SKP2 status, we next examined the activation of Bcr-Abl in CML cells stably expressing control shRNA or shUSP10 with or without HA-SKP2 plasmids. We found that overexpression of SKP2 significantly rescued the activity of Bcr-Abl from inhibition by shUSP10 (Fig. 5a). Consistently, CML cell proliferation inhibited by shUSP10 was reversed by overexpression of SKP2 (Fig. 5b, c). Furthermore, the introduction of WT SKP2 but not inactive mutant SKP2 (S72A)^45^ significantly rescued the downregulation of K63-linked ubiquitination and inactivation of Bcr-Abl resulting from USP10 inhibition (Fig. 5d), suggesting that S72 of SKP2 is critical to the K63-linked ubiquitination and the activation of Bcr-Abl. Taken together, silence or inhibition of USP10 could inactivate Bcr-Abl and displayed SKP2-dependent anti-CML effects.

### Loss or inhibition of USP10 suppresses the growth of CML in vivo

**Fig. 6:**
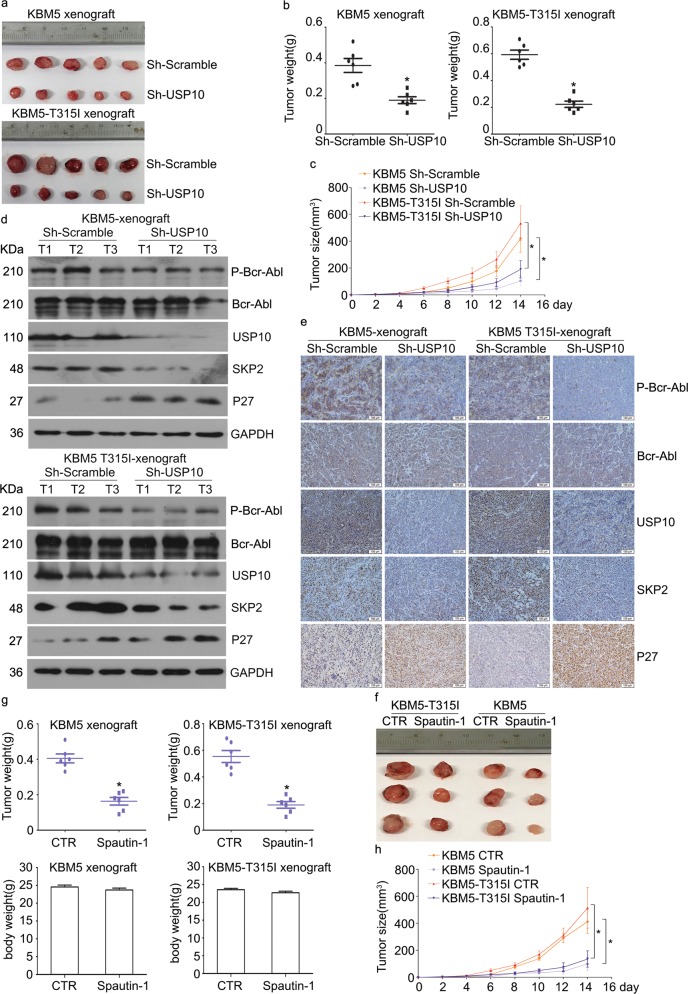
Loss or inhibition of USP10 suppresses the growth of CML xenografts. **a** KBM5 and KBM5 T315I xenografts stably expressing USP10 shRNA or control shRNA were cultured on BALB/c nude mice for 2 weeks. Xenograft images are shown. **b**, **c** Tumor weight and tumor size were recorded. Summarized data are shown. *P < 0.05 versus Sh-Scramble. **d** Western blot assay was used to detect the protein level of P-c-Abl, c-Abl, USP10, SKP2, and p27 of indicated xenografts. **e** Immunohistochemistry staining assay was used to detect the protein expression and localization of p-c-Abl, c-Abl, USP10, SKP2, and p27 of indicated xenografts. Representative images were shown at a magnification of 200. **f** KBM5 and KBM5 T315I xenografts were cultured on BALB/c nude mice and treated with Spautin-1 for 2 weeks. Xenograft images are shown. **g**, **h** Tumor weight, body weight, and tumor size were recorded and summarized. *P < 0.05 versus vehicle control

We next wondered whether loss or inhibition of USP10 suppresses the growth of CML xenografts in vivo. We observed that tumor weight and volumes in the USP10 knockdown group were significantly decreased compared with the scramble group (Fig. 6a–c). Meanwhile, tumor weight and volumes, but not the body weight of mice, in the USP10 inhibition group were also significantly reduced, compared with the vehicle group (Fig. 6f–h). Western blot assays showed that the expression of SKP2 and activation of Bcr-Abl in the xenografts stably expressing USP10 shRNA were significantly reduced, compared with the Scramble xenografts (Fig. 6d). Furthermore, the immunostaining results confirmed that the expression of SKP2 and activation of Bcr-Abl in the xenografts stably expressing USP10 shRNA were significantly reduced compared with the Scramble group (Fig. 6e). These results collectively suggest that pharmacological or genetic inhibition of USP10 is effective for CML treatment in vivo.

### USP10 and SKP2 were upregulated in CML patients

**Fig. 7:**
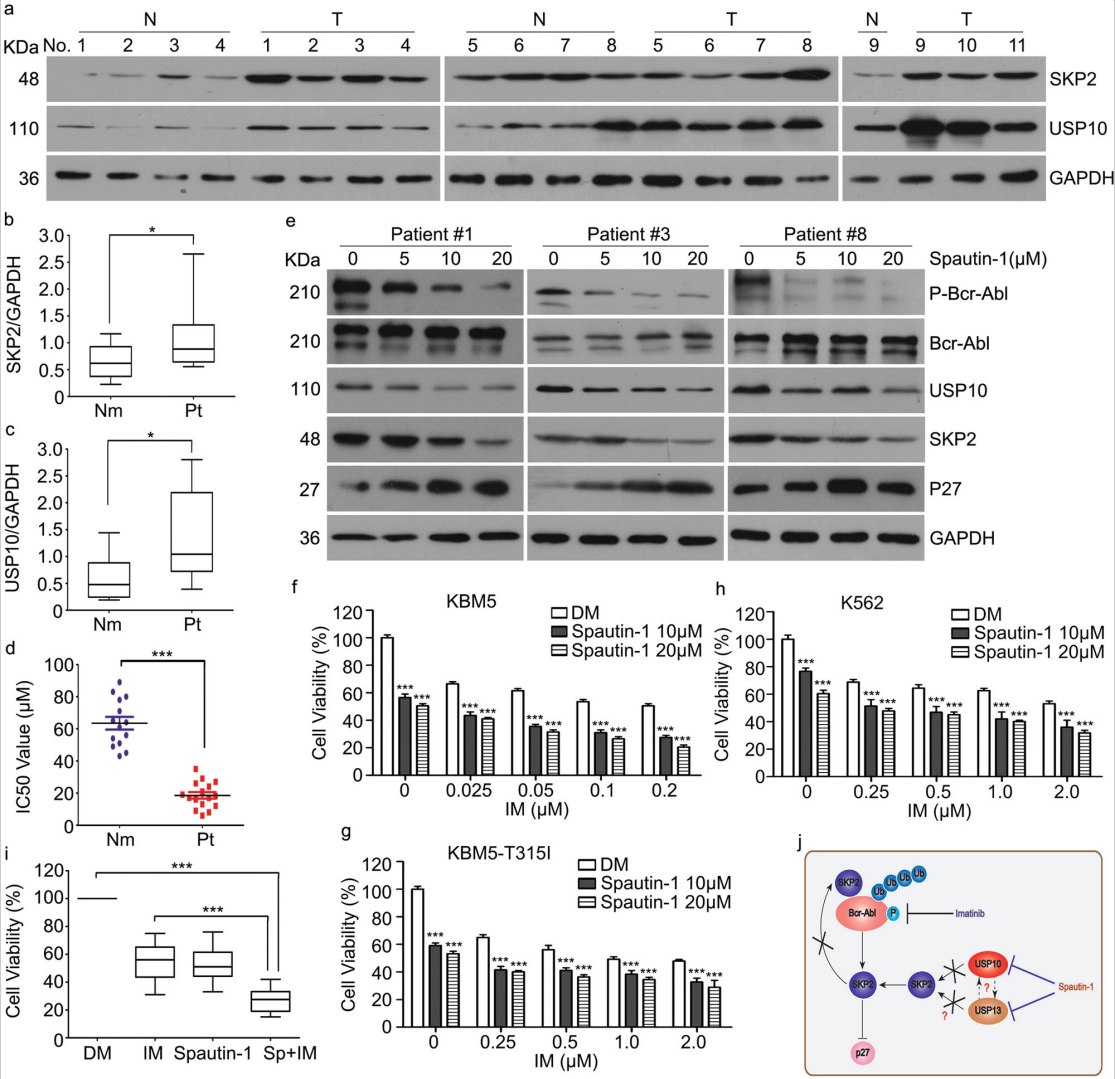
USP10 and SKP2 were upregulated in primary monocytes from CML patients. **a** Western blot assay for USP10 and SKP2 in primary monocytes from humans with or without CML. **b**, **c** Summary of the protein levels of USP10 and SKP2 are shown. *P < 0.05 versus healthy volunteer control. **d** Cell viability of monocytes from CML patients or healthy volunteers exposed to various concentrations of Spautin-1. IC_50_ values were shown. ***P < 0.05 versus healthy volunteer control. **e** Western blot analyses for changes in p-c-Abl, c-Abl, USP10, SKP2 and p27 in primary monocytes from CML patients in response to the indicated dose of Spautin-1 for 48 h. **f**–**i** Cell viability of CML cells and primary monocytes from CML human in response to treatment of Spautin-1combined with or without IM for 24 h. ***P < 0.05 versus vehicle control or IM treatment. **j** A proposed mechanism for USP10 to regulate the SKP2 level and activation of Bcr-Abl

To determine the clinical relevance of regulation of SKP2 by USP10, we performed western blot analysis of USP10 and SKP2 on primary monocytes from 11 patients with CML. We found that USP10 and SKP2 were notably upregulated in patients with CML, compared with healthy controls (Fig. 7a–c). To further determine whether USP10 could be a druggable target in these patients, we evaluated the ex vivo antineoplastic effect of pharmacological inhibition of USP10 by Spautin-1 on primary monocytes from 16 patients with CML. Notably, Spautin-1 significantly decreased the cell viability of primary monocytes from patients with CML with lower IC_50_ values than those of the control subjects (Fig. 7d). Moreover, pharmacological inhibition of USP10 with Spautin-1 also significantly decreased the activation of the Bcr-Abl-SKP2 axis in these patients (Fig. 7e). To determine whether inhibition of USP10 enhances the efficacy of IM, we examined the effect of Spautin-1 or various concentrations of IM alone or in combination on the cell viability of CML cells and primary monocytes from patients. Notably, Spautin-1 enhanced the anti-tumor effect of IM in CML cell lines and primary monocytes from patients (Fig. 7f–i).

Overall, our study suggests that SKP2 mediates the K63-linked ubiquitination and activation of Bcr-Abl to enhance the Bcr-Abl signaling. Additionally, our study suggests that USP10 is a novel DUB for SKP2 and it may work with USP13, mediating a confronting effect against ubiquitination and degradation of SKP2 and thereby ensuring the high protein level of SKP2 and enhanced Bcr-Abl signaling (Fig. 7j).

## Discussion

There is an urgent need to search for effective measures to overcome acquired resistance to TKIs^46,47^ in CML treatment. Defining molecular mechanisms that govern Bcr-Abl activation or identifying novel targets is warranted. Prior reports had shown that expression of F-box protein SKP2 is upregulated by Bcr-Abl signaling and thereby mediates Bcr-Abl oncogenesis through actions like degrading CDK inhibitor p27. Here, we have demonstrated that SKP2 can in turn interact with Bcr-Abl, mediate K63-linked ubiquitination of Bcr-Abl, and promote its activation, thereby forming a forward feedback loop between Bcr-Abl and SKP2 in CML; we also have discovered that USP10 can serve as a DUB to remove K48-linked ubiquitin moieties from SKP2 and thereby stabilizing SKP2; hence, the Bcr-Abl-SKP2 feed-forward loop can be further amplified by USP10. Discovery of the USP10-SKP2-Bcr-Abl axis not only represents an advance in cell biology in terms of understanding the regulation of Bcr-Abl signaling in the cell, provides new insight into the pathogenic mechanisms governing Bcr-Abl based CML, and identifies new potential drug targets for CML. Indeed, we found that both USP10 and SKP2 proteins were significantly increased in the CML cells directly collected from clinical patients. Moreover, we have also experimentally demonstrated that both pharmacological and genetic inhibition of USP10 are capable of, in a SKP2-depednet manner, suppressing the proliferation of both IM-sensitive and IM-resistant CML cells in cell cultures and in mouse xenograft models.

Consistent with previously well documented role for the Bcr-Abl-SKP2-p27 axis in leukemogenesis and CML progression, we found IM treatment dose-dependently reduced SKP2 protein levels and increased p27 protein levels in KBM5 cells but less so in KBM5-T315I cells (Fig. 1b). The latter is known to be IM-resistant. These data confirm that the expression of SKP2 in these leukemic cells requires the kinase activity of Bcr-Abl. What was unexpected is the findings that SKP2 physically interacts with Bcr-Abl and, more interestingly, inhibition of SKP2 ligase activity with SKP2-C25 or siRNA-mediated SKP2 knockdown can significantly decrease the activation-associated phosphorylation, but not the total protein levels, of Bcr-Abl (Fig. 1c–e). It turns out that the activation of Bcr-Abl appears to need K63-linked ubiquitination and the ligase activity of SKP2 is required for this ubiquitination. This is because K63-linked, but not K48-linked, ubiquitin conjugates immunoprecipitated along with Bcr-Abl were markedly reduced by SKP2 inhibition (Fig. 1h).

Constitutively increased activation of Bcr-Abl is a complicated process of phosphorylation. Indeed, ubiquitination is a critical component of post-translational modification that modulates the proteasomal degradation or biological activity of the substrate proteins. K48-linked ubiquitination of a protein mainly leads to proteasomal degradation, while K63-linked ubiquitination of a protein generally regulates trafficking and biological function^48^. The linkage of K63-linked ubiquitin chains is critical to the activation of Akt signaling and the NF-κB pathway^26,49–51^. Importantly, it has reported that SKP2 is capable of mediating K63-linked ubiquitination and activation of kinases such as AKT and LKB1. Here our data suggest that SKP2 mediates the K63-linked ubiquitination and activation of Bcr-Abl, unraveling a new mechanism enhancing the Bcr-Abl signaling and making it more important to better understand the regulation of SKP2 protein expression in the cell.

DUBs are catalytic enzymes which recognize and remove ubiquitin moieties from their specific substrates^31^. Through confronting ubiquitination, DUBs regulate most cellular processes and gradually emerge as novel targets for cancer therapy^52^. SKP2 protein levels are frequently increased in CML cells but this increase is not always associated with an upregulation of its mRNA;^42,43^ hence, reduced SKP2 protein degradation may be in play. This observation prompted us to investigate SKP2-associated DUBs. USP13 is a known DUB of SKP2 and regulates the CDH-SKP2-p27 axis via deubiquitinating and thereby stabilizing SKP2^44^. The present study uncovers that USP10 is another critical DUB that deubiquitinates SKP2 and suppresses the ubiquitin-dependent proteasomal degradation of SKP2, which is compellingly supported by multiple lines of evidence: (1) our Co-IP experiments revealed compelling interaction of endogenous SKP2 with not only USP13 but also USP10 in KBM5-T315I and KBM5 cells (Fig. 2a, c); (2) the interaction of overexpressed SKP2 with overexpressed USP13 or USP10 was also confirmed in HEK293T cells but the USP10 missing the N-terminal 205 amino acid residues failed to bind to SKP2 (Fig. 2d, e); (3) overexpression of either USP13 or USP10 led to a higher SKP2 protein level and decreased p27 but overexpression of a DUB activity-disabled USP10 failed to do so (Fig. 2f, g); (4) shRNA-mediated knockdown or pharmacological inhibition of USP10 with Spautin1 remarkable decreased SKP2 and phosphorylated Bcr-Abl (but not total Bcr-Abl) but increased p27 in multiple CML cell lines (Fig. 2h, Fig. 3a); (5) CHX chase assays showed that shortened the halflife of SKP2 protein (Fig. 4a, c); and (6) both pharmacological and genetic inhibition of USP10 could markedly increase K48-linked ubiquitinated SKP2 (Fig. 4e, g). Therefore, we submit that USP10 and USP13 may co-stabilize SKP2, leading to the high protein level of SKP2, which enhances the activation of Bcr-Abl signaling and eliminates p27 to disrupt cell cycle control in CML cells. Based on this newly identified USP10-SKP2-Bcr-Abl pathway in leukemogenesis, inhibition of USP10 is expected to be efficacious in treating CML and this study has further provided strong in vitro and in vivo evidence to support this proposition.

A highly significant implication of our discovery of the USP10-SKP2-Bcr-Abl pathway is its provision of a new strategy to overcome the resistance to TKIs caused by T315I mutation. T315I mutation prevents TKIs such as IM from docking at Bcr-Abl. USP10 inhibitors such as Spautin-1 do not require docking Bcr-Abl for their inhibition of Bcr-Abl signaling and downstream events. We showed that loss or inhibition of USP10 not only suppressed the proliferation of imatinib-sensitive and imatinib-resistant cells in vitro but also inhibited the growth of imatinib-sensitive and imatinib-resistant xenografts in vivo. Further supporting the clinical translational potential for USP10 inhibition in patients with CML, we observed that the protein levels of both USP10 and SKP2 in these patients were significantly increased (Fig. 7a–c) and primary monocytes from CML patients were more sensitive to the treatment of Spautin-1, compared with those from normal volunteers (Fig. 7d). Different from what we found in CML cells, USP10 was frequently downregulated and correlated with the p53 protein level in renal cell carcinomas^34^. In agreement with our results, SKP2 was shown to be overexpressed in multiple carcinomas, including CML. Consistent with a previous study^53^, we found that Spautin-1 enhanced the sensitivity of IM-resistant CML cells and primary monocytes from CML patients to IM.

In summary, we have uncovered the USP10-SKP2-Bcr-Abl axis in the genesis and progression of CML, which provides novel insight into the dysregulation of cell cycle control. The present study also has identified targeting USP10 as a potentially practicable treatment strategy for CML, even for IM-resistant CML such as T315I-CML.

## Materials and methods

### Materials

Spautin-1 (S7888), Imatinib (S1026), and MG132 (S2619) were from Sellectchem (Houston, TX, USA). SKP2-C25 (M60136) was from Xcessbio Biosciences, Inc. (San Diego, CA). MTS reagent (G3582) was from Promega Corporation (Madison, WI, USA). Co-IP assay kit (14311D) was from Life Technologies (Carlsbad, CA). Antibodies: anti-Ubiquitin (#3936), anti-K63-Ubiquitin (#12930), anti-K48-Ubiquitin (#12805), anti-USP10 (#8501), anti-USP1 (#8033), anti-USP2 (#8036), anti-USP7 (#4833), anti-USP8 (#11832), anti-USP14 (#11931), anti-USP15 (#66310), anti-USP18 (#4813), anti-UCHL1 (#13179), anti-UCHL3 (#8141), anti-CYLD (#8462), anti-A20 (#5630), anti-SKP2 (#2652), anti-p27 (#3686), anti-FLAG (#14793), anti-HA (#3724), anti-phospho-c-Abl(Y245) (#2861), anti-c-Abl (#2862), anti-phospho-STAT5 (#9359), anti-STAT5 (#25656), anti-phospho-Crkl (#3181) and anti-Crkl (#38710) (Cell Signaling Technology, Beverly, MA, USA); anti-UCHL5 (ab124931), anti-USP13 (ab109264) (Abcam, Cambridge, MA); anti-GAPDH (BS60630) (Bioworld Technology, Inc., Louis Park, MN, USA).

### Cell lines and blood samples

KBM5 cell line was generated from a patient with CML^54^. KBM5-T315I cell line was derived from KBM5 and established as previously reported^22^. KBM5 cells were cultured in IMDM (Gibco) with 10% fetal bovine serum in 5% CO_2_ at 37 °C. KBM5-T315I cells were cultured in the same medium in the presence of 1 μM imatinib. K562 was from ATCC and cultured in RPMI 1640 medium with 10% fetal bovine serum in 5% CO_2_ at 37 °C.

Peripheral blood samples were kindly donated from and with the permission of 16 CML patients and 14 healthy volunteers. Blood samples of patients with CML were obtained from discarded material utilized for routine laboratory tests at the Department of Hematology, Guangzhou First Municipal People’s Hospital, Guangzhou Medical University (Guangzhou, China). The study is approved by the ethics committee of Guangzhou Medical University. Mononuclear cells were isolated according to a previous report^23^.

### Cell viability assay

Cell viability was detected using MTS assay (CellTiter 96Aqueous One Solution reagent) as we reported previously^55^. Briefly, 2 × 10^4^ cells in 100 μl of KBM5, KBM5-T315I, and K562 were seeded in 96-well plate and treated with SKP2-C25/Spautin-1 or shRNAs for various length of time. MTS reagent was added to each well at indicated time. The absorbance of density of each well was read for 3 h incubation at 490 nm with a microplate reader (Sunrise, Tecan, Mannedorf, Switzerland).

### Cell cycle analysis

This assay was performed as previously reported^30^. In brief, CML cells were exposed to Spautin-1 or USP10 shRNA for 48 h. Cells were collected and then washed with cold PBS for three times, and then resuspended with 500 μl PBS plus 2 ml 70% ethanol at 4 °C for 12 h. Cells were then centrifuged and washed with cold PBS again, followed by 50 μg/mL PI, 100 μg/mL RNase A, Triton X-100 complex incubation for 0.5 h at 4 °C. Flow cytometry was used to analyze the stained cells.

### Lentivirus shRNA transfection

Lentivirus (pLent-4in1shRNA-GFP) containing a pool of 4 target-specific shRNAs to knockdown the expression of USP10/SKP2 or control shRNAs and lentivirus (pLent-EF1a-FH-CMV-GP) containing SKP2-CDS, SKP2 (S72A)/control vector were constructed and purchased from VigeneBio (Shandong, China). CML cells were transfected and selected as we previously described^30^. In brief, medium containing lentiviruses and polybrene (5 µg/ml; Santa Cruz, CA, USA) was added at a multiplicity of infection of 10 and mixed with the cells. For selecting stably-transfected cells, we proceeded puromycin selection by replacing fresh medium containing puromycin (Santa Cruz, CA, USA) at the concentration of 2 µg/ml.

### Plasmids and transfection

The plasmids harboring the expressing cassette of USP10, USP10(C424A), or USP13 (all fused with a FLAG-tag at their C-terminal) and the control vector were purchased from VigeneBio (Shandong, China). Exponentially growing cells were seeded in six-well plate for 24 h respectively. Cells were transfected with plasmid USP10, USP10 (C424A), USP13 or control vector mixed with appropriate Lipofectamine 2000 reagent (Life Technologies). Transfected cells were cultured for 48 h before further analyses.

### siRNA transfection

Transfection of siRNAs was performed as we previously reported. In brief, HeLa cells were randomly seeded in 60 mm dishes for 24 h. RPMI opti-MEM(Gibco), lipofectamine RNAiMax (Invitrogen) reagent and siRNAs (Santa Cruz, CA) targeting human USP10, USP13, USP14, USP7, UCHL5 siRNAs or control siRNAs (non-specific sequences) mixtures were prepared respectively. After incubation for 15 min, the mixtures was added in each group. Cells were cultured for 48 and 72 h for further analysis.

### Analysis of protein expression and interaction

Protein interaction (Co-IP) analysis was performed as described previously^30^. Briefly, antibodies and dynabeads (Invitrogen) mixtures were incubated on a rotator at room temperature for 16 h. Protein extracts were then added in the antibodies-beads mixtures and incubated on a rotator. After incubation and rotation at 4 °C for 1–2 h, the antibodies-prays mixtures were washed with PBS-T for three times. Mixtures were then suspended with appropriate SDS loading buffer and separated from dynabeads. Western blot was used to analyze protein expression as described previously^55^.

### Animal study

Nude Balb/c mice were purchased from animal center of Guangzhou University of Chinese Medicine and bred and housed at the animal center of Guangzhou Medical University in accordance with ethical treatment of animals. The xenograft models were prepared as previously reported^22^. Briefly, 2 × 10^7^ CML cells stably expressing USP10 shRNA or control shRNA were inoculated subcutaneously on the flanks of 5- to 6-week-old male nude mice. After inoculation for 3 days, the mice successfully bearing xenografts were observed for 14 days before sacrificed for pathological analyses. Besides, 2 × 10^7^ KBM5-wt and KBM5-T315I CML cells were inoculated subcutaneously on the flanks of 5- to 6-week-old male nude mice. After inoculation for 3 days, the mice successfully bearing xenografts were treated with either Spautin-1 (20 mg/kg/day) or vehicle for a total of 14 days. The size of xenografts were measured and calculated as previously reported^22^.

### Immunohistochemical staining

Xenografts were fixed with Formalin and sectioned according to standard techniques. MaxVision kit (Maixin Biol) was used to immunostain xenograft sections (4 μm) according to the manufacturer’s instructions. Primary antibodies were against P-c-Abl, c-Abl, USP10, SKP2, and p27. MaxVisionTM reagent was added on the slide in 50 μl. Brown was developed with 0.05% DAB and 0.03% H_2_O_2_ in 50 mM Tris-HCl (pH 7.6), and the slides were counterstained with hematoxylin. The intensity of brown staining on tissues indicates the expression of proteins. A negative control for each antibody was also included for each xenograft specimen by substituting the primary antibody with pre-immune rabbit serum.

### Statistical analysis

Data are presented as mean ± SD from three independent experiments where applicable. To determine statistical probabilities, Unpaired Student’s *t*-test or one way ANOVA is used where appropriate. Statistical analysis was performed by GraphPad Prism5.0 software (GraphPad Software) and SPSS 16.0. A *p* value < 0.05 was considered statistically significant.

## Supplementary information

Supplementary Information

## Supplementary information

**Supplementary Information** accompanies the paper at (10.1038/s41421-019-0092-z).

## References

[1] Deininger MW, Goldman JM, Melo JV (2000). The molecular biology of chronic myeloid leukemia. Blood.

[2] Ren R (2005). Mechanisms of BCR-ABL in the pathogenesis of chronic myelogenous leukaemia. Nat. Rev. Cancer.

[3] Danial NN, Rothman P (2000). JAK-STAT signaling activated by Abl oncogenes. Oncogene.

[4] Gesbert F, Sellers WR, Signoretti S, Loda M, Griffin JD (2000). BCR/ABL regulates expression of the cyclin-dependent kinase inhibitor p27Kip1 through the phosphatidylinositol 3-Kinase/AKT pathway. J. Biol. Chem..

[5] Li S (2007). Src kinase signaling in leukaemia. Int. J. Biochem. Cell. Biol..

[6] Lugo TG, Pendergast AM, Muller AJ, Witte ON (1990). Tyrosine kinase activity and transformation potency of bcr-abl oncogene products. Science.

[7] Raitano AB, Halpern JR, Hambuch TM, Sawyers CL (1995). The Bcr-Abl leukemia oncogene activates Jun kinase and requires Jun for transformation. Proc. Natl Acad. Sci. USA.

[8] Agarwal A (2008). Absence of SKP2 expression attenuates BCR-ABL-induced myeloproliferative disease. Blood.

[9] Andreu EJ (2005). BCR-ABL induces the expression of Skp2 through the PI3K pathway to promote p27Kip1 degradation and proliferation of chronic myelogenous leukemia cells. Cancer Res..

[10] Chen JY, Wang MC, Hung WC (2011). Bcr-Abl-induced tyrosine phosphorylation of Emi1 to stabilize Skp2 protein via inhibition of ubiquitination in chronic myeloid leukemia cells. J. Cell. Physiol..

[11] Airiau K (2012). ABT-737 increases tyrosine kinase inhibitor-induced apoptosis in chronic myeloid leukemia cells through XIAP downregulation and sensitizes CD34(+) CD38(−) population to imatinib. Exp. Hematol..

[12] Amarante-Mendes GP (1998). Bcl-2-independent Bcr-Abl-mediated resistance to apoptosis: protection is correlated with up regulation of Bcl-xL. Oncogene.

[13] Soliera AR (2012). Gfi-1 inhibits proliferation and colony formation of p210BCR/ABL-expressing cells via transcriptional repression of STAT 5 and Mcl-1. Leukemia.

[14] Johnson JR (2003). Approval summary: imatinib mesylate capsules for treatment of adult patients with newly diagnosed philadelphia chromosome-positive chronic myelogenous leukemia in chronic phase. Clin. Cancer Res..

[15] Savage DG, Antman KH (2002). Imatinib mesylate--a new oral targeted therapy. N. Engl. J. Med..

[16] Kantarjian HM, Talpaz M, Giles F, O’Brien S, Cortes J (2006). New insights into the pathophysiology of chronic myeloid leukemia and imatinib resistance. Ann. Intern. Med..

[17] Kaur P (2007). Nilotinib treatment in mouse models of P190 Bcr/Abl lymphoblastic leukemia. Mol. Cancer.

[18] Morinaga K, Yamauchi T, Kimura S, Maekawa T, Ueda T (2008). Overcoming imatinib resistance using Src inhibitor CGP76030, Abl inhibitor nilotinib and Abl/Lyn inhibitor INNO-406 in newly established K562 variants with BCR-ABL gene amplification. Int. J. Cancer.

[19] Talpaz M (2006). Dasatinib in imatinib-resistant Philadelphia chromosome-positive leukemias. N. Engl. J. Med..

[20] Chen X (2014). Anti-rheumatic agent auranofin induced apoptosis in chronic myeloid leukemia cells resistant to imatinib through both Bcr/Abl-dependent and -independent mechanisms. Oncotarget.

[21] Shah N. P. (2005). Loss of Response to Imatinib: Mechanisms and Management. Hematology.

[22] Shi X (2014). Gambogic acid induces apoptosis in imatinib-resistant chronic myeloid leukemia cells via inducing proteasome inhibition and caspase-dependent Bcr-Abl downregulation. Clin. Cancer Res..

[23] Shi X (2009). Triptolide inhibits Bcr-Abl transcription and induces apoptosis in STI571-resistant chronic myelogenous leukemia cells harboring T315I mutation. Clin. Cancer Res..

[24] Nakayama K (2004). Skp2-mediated degradation of p27 regulates progression into mitosis. Dev. Cell.

[25] Huang H (2005). Skp2 inhibits FOXO1 in tumor suppression through ubiquitin-mediated degradation. Proc. Natl Acad. Sci. USA.

[26] Chan CH (2012). The Skp2-SCF E3 ligase regulates Akt ubiquitination, glycolysis, herceptin sensitivity, and tumorigenesis. Cell.

[27] Lin HK (2010). Skp2 targeting suppresses tumorigenesis by Arf-p53-independent cellular senescence. Nature.

[28] Hershko DD (2008). Oncogenic properties and prognostic implications of the ubiquitin ligase Skp2 in cancer. Cancer.

[29] Lin HK (2009). Phosphorylation-dependent regulation of cytosolic localization and oncogenic function of Skp2 by Akt/PKB. Nat. Cell Biol..

[30] Liao Y (2017). Proteasome-associated deubiquitinase ubiquitin-specific protease 14 regulates prostate cancer proliferation by deubiquitinating and stabilizing androgen receptor. Cell Death Dis..

[31] Huang H (2016). Two clinical drugs deubiquitinase inhibitor auranofin and aldehyde dehydrogenase inhibitor disulfiram trigger synergistic anti-tumor effects in vitro and in vivo. Oncotarget.

[32] Draker R, Sarcinella E, Cheung P (2011). USP10 deubiquitylates the histone variant H2A.Z and both are required for androgen receptor-mediated gene activation. Nucleic Acids Res..

[33] Faus H, Meyer HA, Huber M, Bahr I, Haendler B (2005). The ubiquitin-specific protease USP10 modulates androgen receptor function. Mol. Cell. Endocrinol..

[34] Yuan, J., Luo, K., Zhang, L., Cheville, J. C. & Lou, Z. USP10 regulates p53 localization and stability by deubiquitinating p53. *Cell* **140**, 384–396 (2010). 10.1016/j.cell.2009.12.032 PMC2820153 20096447

[35] Lin Z (2013). USP10 antagonizes c-Myc transcriptional activation through SIRT6 stabilization to suppress tumor formation. Cell Rep..

[36] Deng M (2016). Deubiquitination and Activation of AMPK by USP10. Mol. Cell.

[37] Weisberg EL (2017). Inhibition of USP10 induces degradation of oncogenic FLT3. Nat. Chem. Biol..

[38] Chan CH (2013). Pharmacological inactivation of Skp2 SCF ubiquitin ligase restricts cancer stem cell traits and cancer progression. Cell.

[39] Brasher BB, Van Etten RA (2000). c-Abl has high intrinsic tyrosine kinase activity that is stimulated by mutation of the Src homology 3 domain and by autophosphorylation at two distinct regulatory tyrosines. J. Biol. Chem..

[40] Hantschel O, Superti-Furga G (2004). Regulation of the c-Abl and Bcr-Abl tyrosine kinases. Nat. Rev. Mol. Cell Biol..

[41] Chen Q., Xie W., Kuhn D. J., Voorhees P. M., Lopez-Girona A., Mendy D., Corral L. G., Krenitsky V. P., Xu W., Moutouh-de Parseval L., Webb D. R., Mercurio F., Nakayama K. I., Nakayama K., Orlowski R. Z. (2008). Targeting the p27 E3 ligase SCFSkp2 results in p27- and Skp2-mediated cell-cycle arrest and activation of autophagy. Blood.

[42] Oliveira AM, Okuno SH, Nascimento AG, Lloyd RV (2003). Skp2 protein expression in soft tissue sarcomas. J. Clin. Oncol..

[43] Osoegawa A (2004). Regulation of p27 by S-phase kinase-associated protein 2 is associated with aggressiveness in non-small-cell lung cancer. J. Clin. Oncol..

[44] Chen M, Gutierrez GJ, Ronai ZA (2011). Ubiquitin-recognition protein Ufd1 couples the endoplasmic reticulum (ER) stress response to cell cycle control. Proc. Natl Acad. Sci. USA.

[45] Liu J (2011). Beclin1 controls the levels of p53 by regulating the deubiquitination activity of USP10 and USP13. Cell.

[46] Arya D (2017). MiRNA182 regulates percentage of myeloid and erythroid cells in chronic myeloid leukemia. Cell Death Dis..

[47] Pereira WO (2017). BCR-ABL1-induced downregulation of WASP in chronic myeloid leukemia involves epigenetic modification and contributes to malignancy. Cell Death Dis..

[48] Wang G (2012). K63-linked ubiquitination in kinase activation and cancer. Front. Oncol..

[49] Skaug B, Jiang X, Chen ZJ (2009). The role of ubiquitin in NF-kappaB regulatory pathways. Annu. Rev. Biochem..

[50] Yang WL (2009). The E3 ligase TRAF6 regulates Akt ubiquitination and activation. Science.

[51] Yang WL, Wu CY, Wu J, Lin HK (2010). Regulation of Akt signaling activation by ubiquitination. Cell Cycle.

[52] D’Arcy P, Wang X, Linder S (2015). Deubiquitinase inhibition as a cancer therapeutic strategy. Pharmacol. Ther..

[53] Shao S (2014). Spautin-1, a novel autophagy inhibitor, enhances imatinib-induced apoptosis in chronic myeloid leukemia. Int. J. Oncol..

[54] Beran M (1993). Biological properties and growth in SCID mice of a new myelogenous leukemia cell line (KBM-5) derived from chronic myelogenous leukemia cells in the blastic phase. Cancer Res..

[55] Liao Y (2018). Growth arrest and apoptosis induction in androgen receptor-positive human breast cancer cells by inhibition of USP14-mediated androgen receptor deubiquitination. Oncogene.

