# Supplementary material for: USP10 modulates the SKP2/Bcr-Abl axis via stabilizing SKP2 in chronic myeloid leukemia
Source: Cell Discov. 2019 Apr 30;5:24. doi: 10.1038/s41421-019-0092-z (PMC6488640; doi:10.1038/s41421-019-0092-z)
Supplement: Supplementary file 1 — Supplementary Information [file 41421_2019_92_MOESM1_ESM.pdf]

Supplementary Methods

Apoptosis assay

Apoptosis assay kit was from Keygen Company (Nanjing,China) and performed as our previous description[55]. In brief, CML cells were collected post Spautin-1(5,10,20μM) treatment for 48h. Cells were wash with cold PBS for 3 times. Cells were then suspended, incubated in the staining system: 500μl binding buffer plus 5μl PI and 5μl Annexin-V FITC for 30 min. Flow cytometry was used to analyzed the stained cells.

References

55. Liao Y. et al. Growth arrest and apoptosis induction in androgen receptor-positive human breast cancer cells by inhibition of USP14-mediated androgen receptor deubiquitination. *Oncogene* 37,1896-1910(2018).

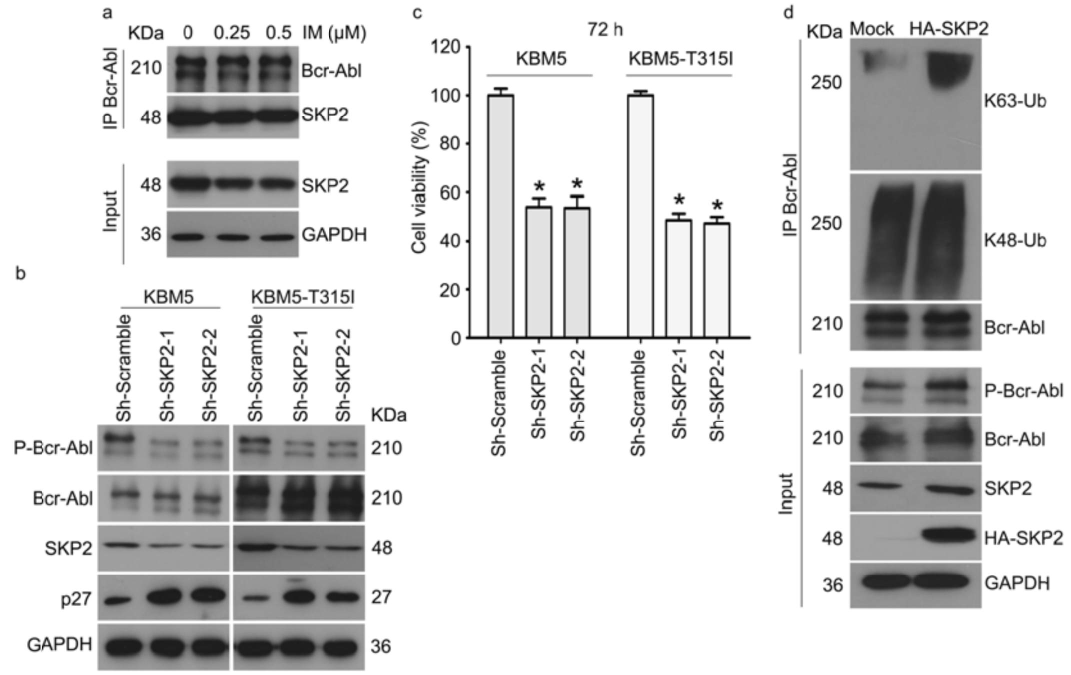

Fig. S1. SKP2 interacts with Bcr-Abl and is required for the activation of Bcr-Abl. a Western blot and Co-IP analysis were used to detect the protein interaction between SKP2 and Bcr-Abl on KBM5 cells post IM treatment for 6h. **b**

Western blot assay and **c** cell viability assay were performed on CML cells stably expressing SKP2 shRNAs. **d** Western blot and Co-IP assay were performed to detect K63/K48-linked ubiquitination of Bcr-Abl on KBM5-T315I cells stably expressing SKP2 or control plasmid.

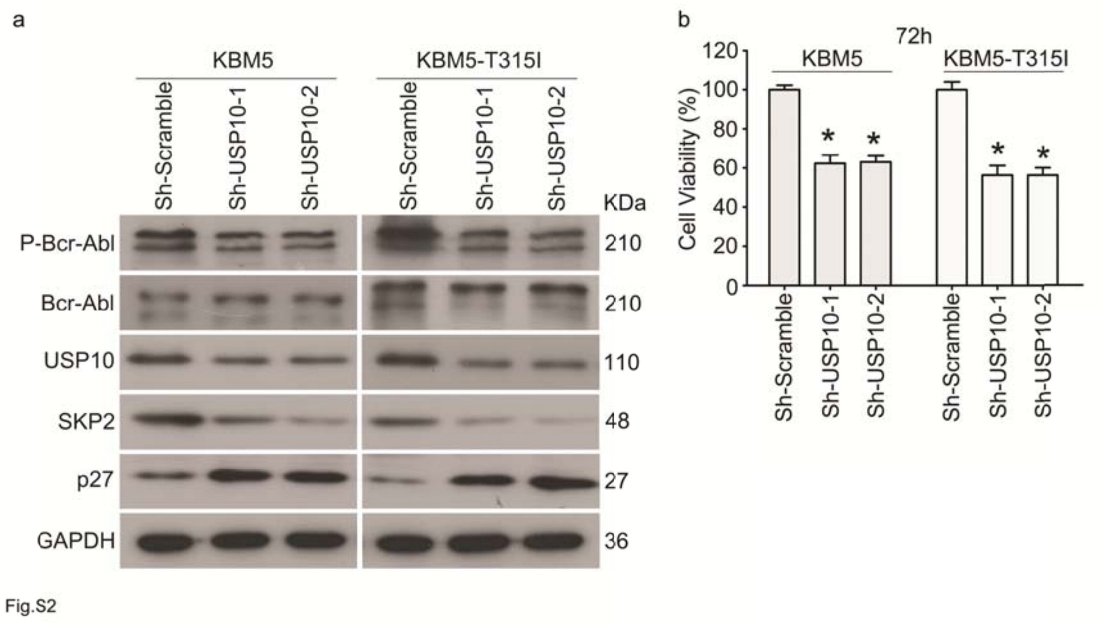

Fig. S2. USP10 interacts with SKP2 and enhances the activation of Bcr-Abl-SKP2. a Western blot assay and **b** cell viability assay were performed on CML cells stably expressing USP10 shRNAs.

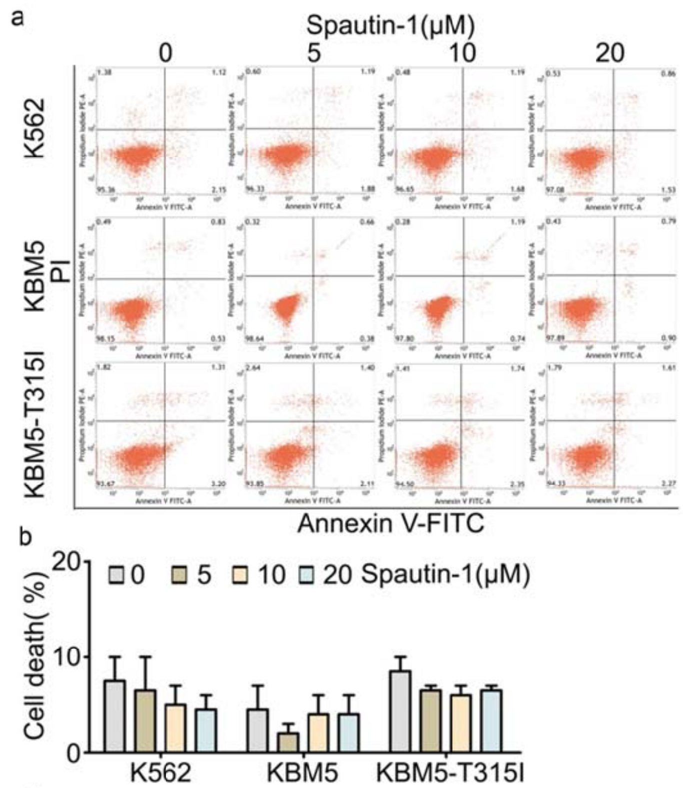

Fig.S3

Fig. S3. USP10 inhibitor Spautin-1 did not induce apoptosis of CML cells. a Representative images shows cell apoptosis of CML cells post Spautin-1 treatment for 48h. **b** Summary data of cell apoptosis in three independent repeats.
